# Investigating Elderly Individuals’ Acceptance of Artificial Intelligence (AI)-Powered Companion Robots: The Influence of Individual Characteristics

**DOI:** 10.3390/bs15050697

**Published:** 2025-05-18

**Authors:** Jing Liu, Xingang Wang, Jiaqi Zhang

**Affiliations:** 1 College of Mechanical and Electrical Engineering, Guangdong University of Petrochemical Technology, Maoming 525000, China; liujing2021@gdupt.edu.cn; 2 Department of Industrial Art, Graduate School of System Design, Tokyo Metropolitan University, Tokyo 1910065, Japan

**Keywords:** artificial intelligence, companion robot, technology acceptance, behaviour intentions, elderly individual

## Abstract

The emergence of AI companion robots is transforming the landscape of elderly care, offering numerous conveniences to senior citizens when their children are not around. This trend is particularly pertinent in ageing societies such as China. Against this backdrop, the present study aims to explore the acceptance of AI companion robots among the elderly from a user-centric perspective. By leveraging insights from existing studies in the literature, we identified three individual characteristic variables—technology optimism, innovativeness, and familiarity—to extend the Artificial Intelligence Device Use Acceptance (AIDUA) model. Subsequently, we developed a conceptual model which was empirically tested through structural equation modelling (SEM) analysis. Our dataset comprised responses from 452 elderly individuals in China. The results revealed that technology optimism and innovativeness were positively associated with performance expectancy and effort expectancy, whereas familiarity inversely predicted perceived risk. Furthermore, emotion was found to be positively influenced by performance expectancy and effort expectancy but negatively impacted by perceived risk. This research extends the AIDUA model within the context of AI companion robots by integrating individual characteristic variables. These findings offer valuable insights for the design and development of companion robots and enrich the domain of Human–Robot Interaction (HRI).

## 1. Introduction

The terminology “companion robots” refers to smart devices endowed with auditory and visual capabilities designed to offer assistive services to individuals with specific needs across various scenarios (Lee et al., 2024). In modern society, many elderly individuals live alone and seldom experience the companionship of their children due to factors such as regional migration, the fast pace of urban life, and the pursuit of independent living. This situation is particularly common in ageing societies. Under such background, companion robots are created as autonomous embodied technologies to fulfil older adults’ needs in both physical and psychological dimensions (Berridge et al., 2023; Gasteiger et al., 2022). On the one hand, these intelligent agents can function as custodial caregivers, assuming a role in housework and healthcare and providing entertainment and surveillance services (Kim et al., 2021). On the other hand, interaction with companion robots can offer social and emotional support and mitigate social isolation among the elderly (Krakovski et al., 2021; Broadbent et al., 2023). A recent article found that companion robots have the potential to address negative emotions of elderly users, such as agitation and anxiety, loneliness and stress (Berridge et al., 2023). For instance, during the coronavirus disease 2019 (COVID-19) pandemic, companion robots were deployed in an ageing care department in the United States to fulfil older people’s socialisation needs (Zilber, 2022).

The rapid advancement of AI technology has made companion robots more attractive to technology giants worldwide. On 8 January 2024, Samsung Electronics unveiled Ballie, an innovative AI companion robot designed to autonomously execute household tasks, manage appliances, and tailor environments for various activities (Samsung Newsroom, 2024). Similarly, LG introduced a companion robot featuring two-legged wheels for enhanced household assistance. By integrating robotics, AI, and multi-modal technologies, this smart agent can learn, understand, and engage in complex communications (Englefield, 2024). In China, the market for companion robots is expansive and flourishing. During the 2024 World Artificial Intelligence Conference (WAIC), Runyi Technology showcased an AI-powered bionic robot specifically aimed at providing emotional companionship for the elderly. This advanced robot can interpret users’ emotional expressions and generate appropriate responses (Ren, 2024). Similarly, iFlytek released the “Ultra Brain 2030” plan in 2023, which aims to develop senior care robots that cover services from healthcare and emotional companionship to home management (Fan, 2024).

China is currently experiencing a rapid ageing process. According to statistics from the World Health Organization (WHO), the population of people aged 60 and above in China is expected to hit 28% by the 2040s, driven by increased life expectancy and decreasing fertility rates (WHO, 2024). This significant demographic shift poses a substantial burden on the social care system (Fan, 2024). At present, the lack of care workers has become the most challenging issue for China’s social care system, which could lead to a senior care crisis. An article in *The Lancet* has also highlighted the concerning shortage of long-term care workers in China (Feng et al., 2020). In this context, the use of companion robots to address the gap in human care workers presents a feasible solution. In addition, the application of companion robots can also benefit individuals. For the elderly, companion robots not only take care of them and improve their physical well-being but also provide emotional support when their children are not around. For family members, these robots provide real-time information on the elderly’s physical status, thereby alleviating the stress and anxiety associated with caring for an elderly family member from a distance (Aldawsari & Chen, 2024). Furthermore, caring for older people often requires a significant amount of time, placing a heavy burden on family caregivers. The deployment of a companion robot can help to lighten this load.

Companion robots have emerged as a hot topic with promising market potential in the situation of global ageing. However, several challenges remain. From a practical perspective, research indicates that senior citizens may be hesitant or even resistant to adopting robotic technologies (Huang & Huang, 2020; Berridge et al., 2023). In Broadbent et al.’s (2018) research, 25 aged participants said that the robot iRobi, a robot offering telemedicine care services, did not bring significant changes in daily lives (Broadbent et al., 2018). Similarly, Berridge et al. (2023) observed that most respondents felt lonelier and more uncomfortable when interacting with companion robots (Berridge et al., 2023). These negative attitudes can be attributed to various factors including poor user experience, lower educational levels, and limited prior usage experience (Huang & Huang, 2020). Revell (2024) also pointed out that social assistive robots can only keep limited engagements and have difficulties in achieving wider user acceptance (Revell, 2024). Additionally, privacy, safety, and ethical concerns may arise from the use of “AI brains” and monitoring sensors in companion robots (Aldawsari & Chen, 2024). From a theoretical perspective, some literature gaps necessitate being filled. While previous studies have explored the acceptance of companion robots among the elderly, few have considered the influence of individual characteristic variables on this acceptance. Moreover, much of the research has focused on traditional technology-acceptance models such as the Technology Acceptance Model (TAM) and the Unified Theory of Acceptance and Use of Technology (UTAUT), with relatively little attention given to the AI Device Use Acceptance (AIDUA) model.

In China and around the world, future societies are expected to experience a significant increase in the number of elderly individuals. As a result, companion robots will be extensively utilised as live-in caregivers. In this context, understanding user acceptance of these smart robots and clarifying the mechanisms underlying their behavioural intentions is crucial, which forms the objective of this research. To address this goal, we proposed two research questions (RQs):

RQ1: What are the key factors influencing elderly users’ acceptance of companion robots? This question will be explored through a comprehensive review of the existing literature.

RQ2: How can a theoretical model be developed to reveal the mechanism of elderly users’ acceptance of companion robots? To answer this, we will employ a structural equation-modelling approach to construct and validate our model.

This study has the following knowledge contribution. From the theoretical viewpoint, this study developed a new model that integrates the Technology Readiness theory and the AIDUA model. In this case, it contributed to the existing literature by extending the well-known AIDUA model and validating the explanatory power of the model under an AI companion robot context. From the practical viewpoint, this study proposed strategies related to AI companion robot design and promotion, which can benefit various stakeholders, including policy makers, robot developers and designers, community workers, and elderly users.

## 2. Literature Review

### 2.1. Companion Robot for the Elderly

**Figure 1:**
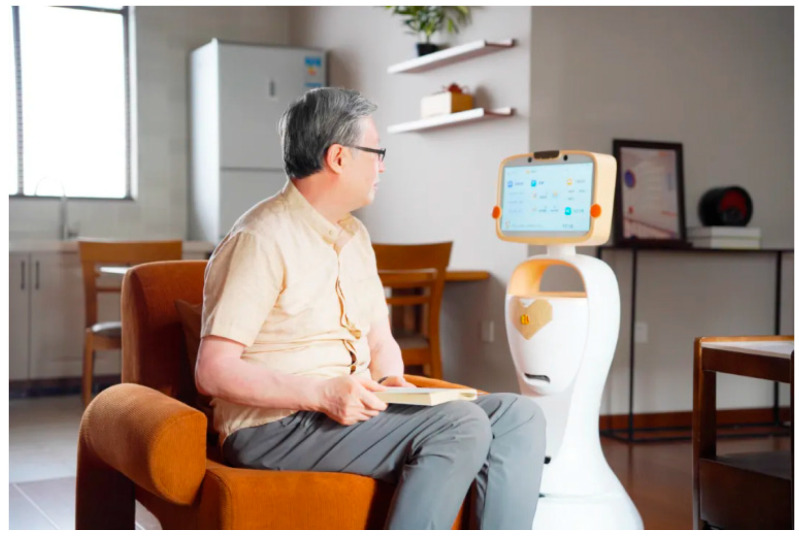
“Xiaoli” elderly companion robot. (Source: https://mp.weixin.qq.com/s?__biz=MzA4MTI0MzgzOQ==&mid=2653316572&idx=3&sn=20676d55b875f3e8db17bd2b7b6e1002&chksm=855f498d08cfa1edbfad6178d1877d5c2b79328d0c7931d954e2440e0349a1860dafd53ca6f9&scene=27) (accessed on 10 February 2025).

**Figure 2:**
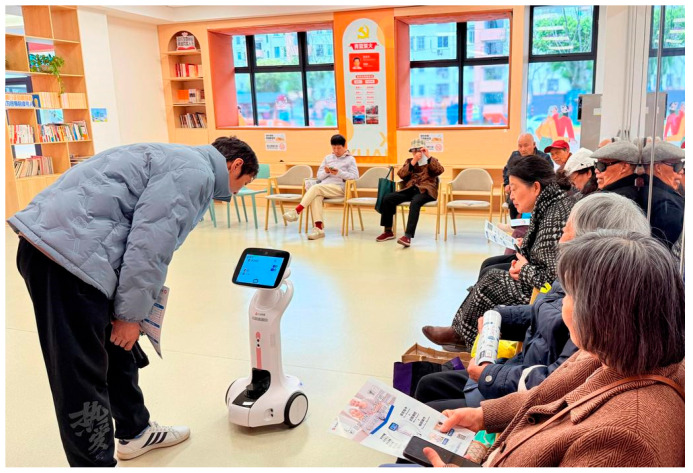
Datou Aliang elderly companion robot (Source: https://baijiahao.baidu.com/s?id=1828252118597248655&wfr=spider&for=pc) (accessed on 10 February 2025).

The utilisation of Agetech among older adults has garnered considerable attention for facilitating healthy ageing (Esmaeilzadeh & Maddah, 2024). As one exemplar of assistive technology, socially assistive robots are designed to encompass a range of functionalities, including lifting, vacuuming, feeding, monitoring, and providing companionship. Fracasso et al. (2022) argued that companion robots were characterised by non-industrial and social assistive (Fracasso et al., 2022). Coghlan et al. (2021) defined companion robots as autonomous social agents equipped with articulated components created to elicit companionable and affective responses, noting that these devices may also perform additional functions (Coghlan et al., 2021). In this study, we defined AI companion robots as a type of non-industrial socially assistive robot embodied with AI technology, which offers assistive services for the elderly in interior spaces, such as home, community, care center, and nursing home (Fracasso et al., 2022; Heerink et al., 2010). Datou Aliang elderly companion robot, “Xiaoli” elderly companion robot from Senlikang Technology, and UBTECH Health Care Robot Series are typical representatives in China. Currently, these companion robots integrate the function of emotional support, health monitoring, emergency alerts, and daily task assistance, improving elderly individuals’ quality of life through comprehensive care services. Figure 1 and Figure 2 demonstrates the application scenario of “Xiaoli” and Datou Aliang elderly companion robot, respectively.

Researchers have investigated how to enhance the acceptability of companion robots to users by leveraging cutting-edge technology. Some efforts have focused on improving the user experience during the adoption of these robots. Some studies have concentrated on optimising human–robot interactions. Rauf and Santoso (2023) created a web-based prototype of a companion robot embodied with a Dual Intent Entity Transformer to grasp users’ intentions more precisely and efficiently. Kok et al. (2024) introduced “Matanya”, a baby-sized companion robot that provides emotional support and care to older adults through the use of facial expression-recognition technology.

The advancement of AI technology has enabled robots to possess a “smart brain”, making them capable companions in recent years (Broadbent et al., 2023). Robots with cognitive AI techniques are one of the representatives of AI-driven robots. These robots can record and learn the conversations with older people and employ psychological skills to facilitate their behaviour change during treatment for chronic illness (Broadbent et al., 2023). Moreover, robots equipped with cognitive AI can recognise and regulate users’ emotions based on the analysis of facial expressions or voice analysis, and offer appropriate feedback (Bertacchini et al., 2023). By using machine learning algorithms, companion robots can adapt and improve their responses based on feedback from the elderly. Also, the emergence of AI-powered speech synthesis allows companion robots to imitate the voices of users’ friends or family members, and then older adults can communicate with these robots in a closer way (C. Wang et al., 2023). Moreover, the application of generative AI technology facilitates easier access to answers or solutions for the elderly through interactions with companion robots at home (Obrenovic et al., 2024).

### 2.2. Elderly’s Acceptance of Companion Robot

Based on the aforementioned definitions, we reviewed the literature exploring individuals’ acceptance of AI-powered “companion robots” or “social assistive” robots for elderly care. It has been found that users exhibit different attitudes towards these intelligent agents. On one hand, senior citizens believe that AI companion robots can enhance both their physical well-being and mental health, and companion robots serve as a supplementary element in their daily lives. In a study by Lee et al. (2024), 18 older adults from South Korea expressed high acceptability for the companion robot “Hyodol”, viewing it as a competent health coach for tasks such as medication reminders and exercise guidance. Notably, the elderly’s attitudes toward companion robots may vary depending on the type of service provided. Previous findings demonstrated that they were receptive to robots for simple assistive tasks, such as reminders, housework, or dialogues. In contrast, they will be reluctant to adopt robots when it concerns intimate physical assistance, such as helping with bathing or toileting (Huang & Huang, 2021).

On the other hand, some older adults are unwilling or even reject to use of companion robots. One main reason is that old people are more likely to fear approaching novel technology. Moreover, they may have a sceptical attitude toward technology, which will make them feel somewhat uncomfortable when interacting with companion robots (Berridge et al., 2023). Prior studies also suggested that companion robots might pose a potential threat to the elderly’s personal well-being, autonomy, and dignity, and they might feel that robots are out of control (Coghlan et al., 2021). This can also lead to people’s resistance to companion robots. Similarly, Aldawsari and Chen (2024) mentioned that intelligent robots may lead to mental disorders (e.g., social isolation) in elder care, as they cannot be a replacement for human caregivers. They also stated that robot caregivers could raise a series of concerns, such as privacy concerns, safety concerns, and ethical concerns, which would be obstacles to adoption intentions (Aldawsari & Chen, 2024).

In information system fields, researchers have examined factors affecting the elderly’s willingness to use companion robots from several aspects. Sun et al. (2024) explored young elderly acceptance from a dual-perspective framework: reasons for their desire to use and reasons for their reluctance. Similarly, Esmaeilzadeh and Maddah (2024) summarised three primary benefits of adopting companion robots—health and wellness improvement, companionship and support, and technological design advantages—alongside three main concerns: digital dependency and social disconnection, information integrity and online resilience, and implementation costs. These authors verified how these factors contribute to the elderly’s willingness to use companion robots. Chi et al. (2023) carried out a comparative analysis to check users’ acceptance of AI-driven service robots in different cultural contexts. They found that several cultural factors, such as uncertainty avoidance, long-term orientation, and power distance, could demonstrate significant moderating effects. Aymerich-Franch and Gómez (2024) found that perceived vulnerability and privacy concerns will reduce people’s acceptance, and prior experience and gender were significant demographic variables related to usage intention. Furthermore, some researchers adopt a comprehensive approach to describe user adoption intentions. For example, Chuah et al. (2021) investigated the impact of combined variables, including human-like attributes, technology-centric features, and consumer characteristics, on users’ acceptance of service robots.

### 2.3. Individual Characteristics in Technology Acceptance

Generally, different people exhibit varying attitudes or performance levels toward a given technology, which means that individual characteristics play a pivotal role in most technology adoption contexts (Venkatesh, 2022). According to Venkatesh’s (2022) research, individuals who exhibit a propensity for risk-seeking behaviour, a high tolerance for uncertainty, and a strong desire for learning are more inclined to adopt AI tools. In addition, some traditional personality traits, such as computer self-efficacy and computer playfulness, are also related to technology adoption. Venkatesh’s (2022) statement echoes the well-known Technology Readiness theory. Technology readiness refers to a mental predisposition that encompasses one’s willingness and enthusiasm to use novel technologies to achieve a particular goal (Parasuraman, 2000). In 2000, Parasuraman introduced the theory of the Technology Readiness Index, which consists of 36 attributes to describe individual differences in technology adoption (Parasuraman, 2000). Parasuraman’s theory can be summarised into four main dimensions: optimism, innovativeness, discomfort, and insecurity. The first two components are contributors, and the last two components are inhibitors. According to Parasuraman and Colby’s exposition, optimism is a view that technology can bring enhanced control, flexibility, and efficiency, innovativeness represents the tendency to be a technology pioneer, discomfort is a belief that technology is out of control or overwhelms users, insecurity describes people’s distrust and doubt of technology (Parasuraman & Colby, 2015). In the Diffusion of Innovation theory, Rogers and Roger also mentioned that innovative people are more likely to develop positive attitudes towards technology (Rogers et al., 2014). The role of individual characteristics on people’s technology adoption willingness has also been validated in the existing literature (Almaiah et al., 2022; Álvarez-Marín et al., 2023). In this regard, we attempted to examine the influence of individual differences on AI comparative robots and added several individual characteristic variables to the proposed model.

### 2.4. AIDUA Model

The AIDUA model served as the theoretical foundation of this study. Developed by Gursoy et al. (2019), AIDUA is a robust model used to predict customers’ intention to adopt AI-powered devices. The AIDUA model suggests that the mechanism of intention to use AI-driven devices can be divided into three stages. In the primary appraisal stage, performance expectancy and effort expectancy are predicted by social influence, hedonic motivation, and anthropomorphism; In the secondary appraisal stage, performance expectancy and effort expectancy will arouse users’ emotions; In the outcome stage, emotion will lead to two adverse behaviour intentions: willingness or objection to adopt a technology. AIUDA integrates Lazarus’s Cognitive Appraisal Theory and cognition–motivation–emotion model, positing that responses stem from emotions arising from multifaceted cognitive evaluations of stimuli (Lazarus, 1991a, 1991b; Gursoy et al., 2019). Huang and Huang (2020) pointed out that companion robots offer value by mentally connecting with humans, which enables them to affect interactions in a meaningful way and foster a sense of companionship. Therefore, AIDUA, with its focus on emotions during the decision-making process, is more appropriate than other theories for understanding the acceptance of companion robots. Since being developed, AIUDA has shown a strong power in explaining people’s readiness to accept the usage of AI facilities, including service robots, autonomous vehicles, various versions of ChatGPT, and GenAI-based financial services (Alsaad, 2023; Chi et al., 2022; Ma & Huo, 2023; Yang & Lee, 2024). Moreover, researchers have extended the original AIDUA to make it more suitable for the situation of their studies. The addition variables include dimensions of anthropomorphic features, novelty value and humanness, trust, and cultural contexts (Li et al., 2024; Ma & Huo, 2023; Chi et al., 2023).

## 3. Hypotheses Development and Research Model

**Figure 3:**
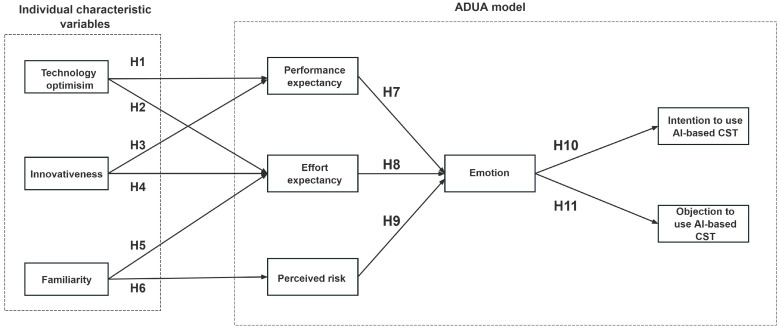
Theoretical model.

According to Venkatesh’s (2022) study, technology-related traits can serve as prerequisites for performance expectancy and effort expectancy. Thus, we selected two individual characteristic variables from Parasuraman’s (2000) Technology Readiness Index Theory and put them as variables in the primary appraisal stage. Familiarity was another variable that represented individuals’ usage experience of AI companion robots. Performance expectancy and effort expectancy, two AIDUA variables, were retained in the second appraisal stage. Moreover, the variable “perceived risk” could be added to be in parallel with these two variables, which reflects the negative side of users’ perceptions during companion robot adoption (Ribeiro et al., 2022). Notably, perceived risk can also echo the inhibitor “discomfort” in the Technology Readiness Index. Finally, three original AIDUA variables, emotion, willingness to use, and objection to use, were retained in the outcome stage. Drawing on insights from the existing literature, a conceptual model was finally proposed, see Figure 3.

### 3.1. Technology Optimism

When individuals believe that a technology will yield positive and potential benefits, they tend to develop favourable attitudes towards it and are more inclined to adopt the technology. This mechanism is known as technology optimism (Álvarez-Marín et al., 2023). Chung et al. (2015) stated that this factor revealed one’s preparedness to adopt a technology. In the context of this study, technology optimism characterises users who have optimistic views regarding the social value and development of AI-enabled companion robots (Y. Liu et al., 2021). In China, where companion robots have not yet become widespread and caregivers remain dominant in the elderly care system, some people express concerns about AI and robotic technologies, fearing privacy losses or unethical issues. However, individuals with an optimistic outlook on AI and robotics are more likely to emphasise the benefits and usefulness of AI companion robots. Academic research supports this notion. For instance, Álvarez-Marín et al.’s (2023) research suggested that technology optimism could drive users’ positive attitude toward using AR techniques. Similarly, Almaiah et al.’s (2022) study indicated that technology optimism was a significant predictor of perceived usefulness and perceived ease of use. Based on the above illustration, we hypothesised that:

**H1.**  *Technology optimism positively affects performance expectancy.*

**H2.**  *Technology optimism positively affects effort expectancy.*

### 3.2. Innovativeness

Innovativeness was proposed as another variable that reflects individual differences during technology adoption. Based on the Diffusion of Innovations theory, highly innovative individuals serve as pivotal agents in the diffusion of new technology since they cultivate a favourable attitude towards the technology (Shanmugavel & Micheal, 2022). People with innovativeness are technology pioneers who believe that novel technologies are not complex or exceed their comprehension (Álvarez-Marín et al., 2023). This type of user is more likely to explore and accept new technologies compared with other counterparts in society (Karahanna et al., 1999). In this study, personal innovativeness reflects users’ willingness to experience AI and robotic technology (Jang & Lee, 2018). AI-based companion robots are currently not widely applied in China, so many potential users do not have experience or a deep understanding of this technology. Thereby, users with innovativeness are more likely to try out AI companion robots (Shanmugavel & Micheal, 2022). In addition, there is research affirming the impact of personal innovativeness on attitudes toward new technology. For instance, Álvarez-Marín et al. (2023) found that innovativeness could trigger users’ attitudes toward adopting AR technology. Almaiah et al.’s (2022) research revealed that technology innovativeness could contribute to perceived ease of use and perceived usefulness. The relationship between personal innovativeness and perceived usefulness has also been validated in Shanmugavel and Micheal’s (2022) study (L. Wang et al., 2024). Thus, the following hypotheses were formulated:

**H3.**  *Innovativeness positively affects performance expectancy.*

**H4.**  *Innovativeness positively affects effort expectancy.*

### 3.3. Familiarity

Familiarity refers to users’ understanding of a technology or product that relies on their prior experience, knowledge, and learning (J. Liu et al., 2023). Alba and Hutchinson (1987) argued that familiarity was based on people’s accumulated experience of a product or service. In this study, familiarity describes an individual’s usage experience or comprehension of companion robots driven by AI technology. Generally, customers who are familiar with companion robots have a greater tendency to accept a new technology or product, particularly for older adults (Y. Liu et al., 2021). As we know, many senior citizens are unwilling or even fear accepting a technology that is closely related to their daily lives. Familiarity was confirmed as a critical prerequisite for technology adoption. In Choi’s (2020) study, familiarity could trigger customers’ perceived ease of use and perceived usefulness with food delivery phone applications. The association between familiarity and perceived risk has also been discussed in many scenarios. For instance, Nepomuceno et al. (2014) stated that for users who are knowledgeable about the desired product, familiarity could minimise risk perceptions during online purchases. Chang et al. (2016) argued that product familiarity could potentially lower customers’ perceived risk on a shopping website. Shavit et al. (2016) highlighted that unfamiliarity with a particular technology could augment perceived risk and related harm. In the present study, we believe that users who have prior knowledge or experience of AI-based companion robots will enjoy more ease of use and have fewer risk perceptions toward AI-enabled companion robots. Thus, the following hypothesis was put forward:

**H5.**  *Familiarity positively affects effort expectancy.*

**H6.**  *Familiarity negatively affects perceived risk.*

### 3.4. Performance Expectancy

Performance expectancy is a popular and critical variable in many well-known technology adoption theories, such as TAM, UTAUT, UTAUT2, and AIDUA. Gursoy et al. (2019) described performance expectancy as to what extent users believe that adopting technology will aid them in completing a task in the AIDUA model. In Nan et al.’s (2022) research, performance expectancy was described as consumers’ perceived utility and benefits of face recognition payment. In the present study, performance expectancy refers to users’ expectations of how using AI-based companion robots can improve their quality of life in many aspects, such as assistance, healthcare, and entertainment. These utilitarian values provided by companion robots make them more acceptable and arouse customers’ positive emotions (Ma & Huo, 2023; Venkatesh et al., 2012). The association between performance expectancy and emotion was derived from the original AIDUA model and has been validated in different technology adoption scenarios. For instance, Ma and Huo (2023) found that performance expectancy could contribute to users’ cognitive attitudes and affective attitudes during ChatGPT adoption. This relationship was also verified in Chi et al. (2022, 2023), and Li et al.’s (2024) research on AI-enabled devices. In this regard, we hypothesised that:

**H7.**  *Performance expectancy positively affects emotion.*

### 3.5. Effort Expectancy

Based on Venkatesh et al.’s (2012) article, we defined effort expectancy as “the degree of ease related to customers’ adoption of AI-based companion robots. This definition aligns with the behavioural-level design aspect of Norman’s Emotional Design theory, which focuses on the functionality and usability of a product or service (Norman, 2004). Moreover, the literature in human–computer interaction fields also highlights that ease of use is closely associated with customers’ willingness. People generally tend to prefer a product that is easy to operate and understand. In contrast, if the robot’s operation is complex and difficult to comprehend, users will develop a resistant attitude. In other words, the simplicity and clarity of a companion robot’s operations and interaction modes directly impact the user’s experience and emotion during adoption. Prior studies have revealed that effort expectancy could affect users’ attitudes towards using various AI-based technologies such as chatbots, hotel service robots, and medical robot AI (Ma & Huo, 2023; Li et al., 2024; Lin et al., 2020). Thus, we believed that effort expectancy would arouse positive emotion and user experience during companion robot usage, and the hypothesis was posed:

**H8.**  *Effort expectancy positively affects emotion.*

### 3.6. Perceived Risk

The factor “perceived risk” was proposed as one of the three pillars that determine the emotion to implement companion robots in this research. For an information system, this factor can lessen the possible utility attached to the technology (Bansal & Gefen, 2010). Prior research defined perceived risk as the “potential for losses (or adverse consequences) an individual perceives in the pursuit of desired outcomes when adopting a technology (Zhao et al., 2025). Kamal et al. (2020) pointed out there were six types of perceived risks: performance, financial, social, psychological, safety, social, and opportunity/time. In this article, perceived risk refers to customers’ perception of uncertainty and potential adverse outcomes caused by AI companion robots. Currently, users have an uncertain attitude toward companion robots for several reasons. First, AI-powered companion robots are not widely applied in China. Many people are unfamiliar with it and do not understand how it works, which is a barrier to adoption (Tizhoosh & Pantanowitz, 2018). Second, several risks caused by companion robots have been reported, including safety concerns, ethical concerns, and privacy loss (Revell, 2024; Aldawsari & Chen, 2024). Third, companion robots may pose a potential risk to users’ well-being, autonomy, and dignity of elderly individuals (Coghlan et al., 2021). In academic fields, previous research has demonstrated a negative effect of perceived risk on behaviour intention (Nan et al., 2022; Seo & Lee, 2021). Moreover, Ribeiro et al. (2022) added perceived risk to the AIDUA model and found that this variable could be a negative trigger of emotion in the context of autonomous vehicles. Building on this, we proposed the hypothesis:

**H9.**  *Perceived risk positively affects emotion.*

### 3.7. Emotion

“Emotion” is an original AIDUA variable, which refers to users’ subjective mental states that cultivate their behavioural actions and assist them in organising their behavior implementations (Gupta, 2024). In the cognitive assessment theory, after the assessment process, people’s emotion will ultimately determine their acceptance of medical service robots (Lazarus, 1991a; Li et al., 2024). In this study, the factor “emotion” describes people’s affective attitudes toward using AI-based companion robots. According to the AIDUA model, emotion can play a two-sided role in affecting individuals’ intentions to adopt AI devices. Some positive emotions, such as relaxation, contentment, and hope, will facilitate users’ acceptance of technology. In contrast, negative emotions, such as boredom, melancholy, and despair, will be an obstacle to technology adoption intentions (Gursoy et al., 2019). Prior literature has revealed bidirectional effects of emotions on people’s intentions to use AI-based service robots, ChatGPT, and medium robots (Chi et al., 2023; Ma & Huo, 2023; Li et al., 2024). Hence, we came up with the hypotheses:

**H10.**  *Emotion positively affects willingness to use AI-based companion robots.*

**H11.**  *Emotion negatively affects objection to the use of AI-based companion robots.*

## 4. Methodology

### 4.1. Study Design

This study employs an observational cross-sectional design to examine causal relationships among key variables in the proposed theoretical model among elderly users or potential users of AI companion robots in China. Using purposive sampling, data were collected via a questionnaire survey between October 2024 and December 2024. Regarding the research ethics, this study referred to the guidelines named “Measures for Ethical Review of Life Science and Medical Research Involving Humans (Article 32, Chapter 3)”, which was launched by the State Council of China. Regarding the statistical method, we carried out an SEM approach for data analysis. Overall, our research adhered to the Strengthening the Reporting of Observational Studies in Epidemiology (STROBE) guidelines for transparent reporting of cross-sectional research.

### 4.2. Questionnaire Development

The questionnaire contained three sections. To begin with, we presented a brief introduction of the survey, which includes three paragraphs: The first paragraph emphasized our research backgrounds and research objective; The second paragraph demonstrates related terminology of this study (e.g., AI companion robots); The third paragraph was about several notices (e.g. informed consent form, ethic declaration) for respondents before the questionnaire survey. Overall, the initial section aims to enable participants to have a fundamental understanding of the questionnaire survey. In the second section, respondents were required to provide a series of demographic information when filling out the questionnaire, including gender, age, educational levels, and family monthly income. Following this was a question that tried to gain participants’ attitudes toward AI-based companion robots. In the third section, a series of questions were set to measure variables in the above-proposed model. These questions were all adapted from prior studies, see Table A1. To be more specific, three items of familiarity were developed by J. Liu et al. (2023), and the scale of technology optimism and innovativeness was revised from (Álvarez-Marín et al., 2023; J. Liu et al., 2023). Moreover, we used the questions from Venkatesh et al.’s (2012) research to measure performance expectancy and effort expectancy. Four items adapted from Esmaeilzadeh’s (2020) study were employed to measure the variable “perceived risk”. Lastly, the items from Lu et al.’s (2019) study were adopted to measure three variables: emotion, willingness to use, and objection to use. Overall, there are 9 questions and 33 sub-questions in total in this part, where each question corresponds to a latent variable and the sub-question corresponds to a measurement item. A five-point Likert scale was employed to assess the observed variables, wherein the integers ranging from “1” to “5” correspond to responses from “strongly disagree” to “strongly agree”, respectively. We used “Wenjuanxing”, a popular questionnaire survey platform, to create the questionnaire.

After the initial version of the questionnaire was developed, we invited six experts in design and bilingual study fields to conduct a group interview. The interview was held through an online meeting in September 2024 to refine the questionnaire. The interview commenced with a presentation introducing the aim of this study and the questionnaire. Subsequently, both the English version of the questionnaire and its translated Chinese version were shared with the experts. Experts were divided into groups based on their expertise to provide targeted feedback: bilingual studies experts focused on identifying regional linguistic disparities, semantic ambiguities, and translation inaccuracies, while design experts evaluated all other issues, such as structures, formats, and usability. After that, we collected all suggestions generated by all experts, and they voted to produce the top priority improvements of the questionnaire. Finally, we modified the questionnaire based on experts’ suggestions, and the revised questionnaire was re-evaluated by experts to ensure alignment with their feedback later. The summary of experts’ suggestions for the questionnaire is shown in Table A2.

### 4.3. Sampling Strategy

The targeted respondents are elderly users or potential elderly users of AI companion robots. To begin with, we set the following standards to screen appropriate informants for this study: First, the informants’ age should be over 60 years old, according to the criteria of age classification by the WHO. Moreover, they should have usage experience or related knowledge of AI-based companion robots. Lastly, they should be Chinese since our research scope was in China. Once the recruitment standards were met, we attempted to find people who were willing to participate in this study through various methods. Our sampling procedure includes an online survey and an offline survey. For the online survey, we tried to recruit respondents through social networking websites or phone applications. Although older adults are not the main users of social media, an increasing number of elderly people are using social media in China recently. Thereby, we attempted to join related interest groups of the elderly on some popular social applications and platforms, such as WeChat, Sina Weibo, and Douban. Then we distributed the questionnaire to these online platforms and waited for replies. For the offline survey, we went to local senior centres and senior communities to look for appropriate informants.

### 4.4. Data Analysis

Once all the data were collected and recorded, we carried out an SEM analysis that contained the following steps. First, a descriptive analysis was conducted to show the demographic information of respondents, including gender, age, education, and income. Second, we conducted a confirmatory factor analysis (CFA) to check the measurement model through a series of indices, such as composite reliability, convergent validity, discriminant validity, and so on. Finally, the proposed hypotheses were tested during a path analysis. During these steps, the SPSS 25.0 software was employed for descriptive analysis and internal reliability testing, and the MPLUS 8.3 software was utilised for confirmatory factor analysis (CFA) and path analysis. Notably, the SEM analysis in this study belongs to Covariance-Based SEM (CB-SEM). CB-SEM and Partial Least Squares-based SEM (PLS-SEM) are two widely utilised approaches.

## 5. Results

### 5.1. Participants

We collected 452 valid answers during the questionnaire survey. Table 1 shows the demographic information of respondents. This study involved 452 participants, comprising 250 males and 202 females, with their ages categorised into five levels: 103 individuals aged 60–64, 134 aged 65–69, 134 aged 70–74, 63 aged 75–79, and 18 aged 80 or above. Educational attainment varied, with 89 participants having completed primary school or below, 111 with junior high school education, 119 with high school/technical secondary school qualifications, 90 with a diploma, and 43 possessing an undergraduate degree or higher. Monthly household income levels were distributed as follows: 63 participants earned under 3499 RMB, 135 earned between 3500 to 6999 RMB, 145 earned between 7000 to 10,499 RMB, 83 earned between 10,500 to 13,999 RMB, and 26 earned over 14,000 RMB. This diverse sample provides a comprehensive overview of the demographic characteristics relevant to understanding the factors influencing elderly individuals’ acceptance of AI companion robots.

### 5.2. Measurement Model Results

Table 2 summarises several indices used to evaluate the reliability and validity of the proposed model. It can be concluded that these variables exhibit a satisfactory level of composite reliability, all exceeding 0.7 (Fornell & Larcker, 1981). Additionally, the Standardised Factor Loadings of the constructs ranged from 0.779 to 0.901, while the Average Variance Extracted (AVE) values fell between 0.677 and 0.762, surpassing the recommended threshold of 0.5 (Anderson & Gerbing, 1988). This indicates that the convergent validity of the measurement model meets the required criteria. Subsequently, we compared the square roots of the AVEs for the latent variables (presented on the diagonal in Table 3) with their respective correlation coefficients. The results show that the square roots of the AVEs were higher than the correlation coefficients, thereby confirming appropriate discriminant validity, see Table 3 (Fornell & Larcker, 1981).

Based on a series of indices used to assess the goodness-of-fit of the model, we found that both the measurement model (chi-square/df = 2.058, the root mean square residual (SRMR) = 0.025, the root mean square error of approximation (RMSEA) = 0.048, normed-fit Tucker–Lewis Index (TLI) = 0.946, and comparative-fit index (CFI) = 0.953) and structural model (chi-square/df = 1.388, SRMR = 0.072, RMSEA= 0.029, TLI = 0.965, and CFI = 0.968) had good model fit (Hu & Bentler, 1999).

### 5.3. Structural Model Results

**Figure 4:**
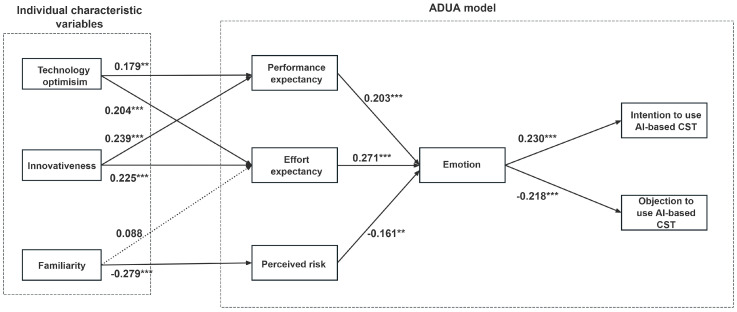
The results of path analysis. Note: ** *p* < 0.01; *** *p* < 0.001. The solid arrow denotes significant relationships, and the dashed arrow denotes non-significant relationships.

The CFA results furnished robust evidence supporting the validity and reliability of the measurement model. After that, we carried out path analysis to gain the result of the structural model, and the results are shown in Table 4 and Figure 4. The path analysis results indicated that ten of the eleven proposed hypotheses were accepted, except H5. Among the ten supported hypotheses, eight of them show high statistical significance (*p* < 0.001), and the remaining two show moderate statistical significance (*p* < 0.01). In the primary appraisal stage, performance expectancy was positively influenced by technology optimism (*p* < 0.01, t = 2.961) and innovativeness (*p* < 0.001, t = 4.060), so H1 and H3 were supported. Effort expectancy was simultaneously affected by two variables: technology optimism (*p* < 0.001, t = 3.674), and innovativeness (*p* < 0.001, t = 3.920); thereby, H2 and H4 were supported. H5 was not supported since familiarity did not contribute to effort expectancy (*p* > 0.05, t = 1.658). Moreover, we observed that familiarity (*p* < 0.001, t = −5.360) was a negative predictor of perceived risk, which indicated that H6 was supported. In the secondary appraisal stage, performance expectancy (*p* < 0.001, t = 3.787), effort expectancy (*p* < 0.001, t = 5.185), and perceived risk (*p* < 0.01, t = −3.409) were all critical drivers of emotion, suggesting that H7, H8, and H9 were supported. Finally, in the outcome stage, emotion could positively affect willingness to use (*p* < 0.001, t = 4.325) and negatively affect objection to use (*p* < 0.001, t = −3.911). Hence, H10 and H11 were supported.

### 5.4. Moderating Effects

Based on the identified path relationships, we conducted a multi-group analysis to examine the moderating effects of gender, age, and education level on the six significant relationships in the primary appraisal stage. The groups were categorised as follows: for gender, male and female; for age, a low-level group (ages 60–64 and 65–69) and a high-level group (ages 70–74, 75–79, and 80 or above); and for education, a low-level group (primary school or below, junior high school) and a high-level group (high school/technical secondary school, diploma, undergraduate or above). The results, presented in Table 5 (gender), Table 6 (age), and Table 7 (education), indicate that there are no significant differences between the different gender, age, or education groups regarding these path relationships. This suggests that the relationships hold consistently across various demographic segments.

## 6. Discussion

The current study proposed a model to reveal the mechanism of the elderly’s acceptance of AI-based companion robots in China. In the primary appraisal stage, three individual characteristic variables were found to be critical determinants. In the second appraisal stage, performance expectancy, effort expectancy, and perceived risk are key drivers of users’ affective attitudes. Finally, affective attitudes could play a two-sided role in determining users’ acceptance of AI companion robots. The results support most proposed hypotheses and demonstrate the explanatory power of the AIDUA model in the context of AI companion robots. We then discussed these theoretical findings and developed corresponding design strategies to enhance people’s willingness to use from different stakeholders’ viewpoints, including companion robot developers, policymakers, designers, and community workers.

Technology optimism was found to serve as a positive predictor of performance expectancy and effort expectancy, which was in accordance with Almaiah et al. (2022) and Álvarez-Marín et al.’s (2023) research. This result suggested that individuals with an optimistic perspective on novel technology are more inclined to emphasise the benefits and utility of AI companion robots. Generally, technology optimists base their emphasis on benefits rather than drawbacks brought by technology, and their optimism comes from a well-informed understanding of the technology. For policymakers, they can initiate massive campaigns that highlight the benefits and practical applications of AI companion robots. These campaigns can be launched through TV programs or social networking platforms, especially those oriented towards older people.

Innovativeness was another individual feature variable that affects performance expectancy and effort expectancy, which could mirror Almaiah et al. (2022), Álvarez-Marín et al. (2023) and Shanmugavel and Micheal’s (2022) findings. This means that technology pioneers are more likely to experience the potential benefits and usability of AI companion robots. For designers, they can incorporate early adopters’ stories and experiences into the product design process. For instance, using narratives from technology pioneers in onboarding tutorials can make the initial learning process more engaging and less daunting for new users. Moreover, the cases or stories of technology pioneers can also be embodied in marketing and promotional materials for AI companion robots.

We observed that familiarity could serve as a negative determinant of perceived risk. This means that users who are familiar with AI companion robots will show fewer concerns, which can echo Nepomuceno et al. (2014), Chang et al. (2016), and Shavit et al.’s (2016) research. At present, AI companion robots are an emerging technology and have a long way to go to become popular and widely accepted. Many people are unfamiliar with AI-powered CSTs, so their concerns will be easily transformed into risk perceptions. On this occasion, it is necessary to improve the elderly users’ familiarity with AI companion robots. For designers, they should introduce the basic functions and benefits of AI companion robots to senior citizens in an easy-to-understand way, for example, through hands-on demonstration. In addition, they can provide training on the operation of AI companion robots to build trust and familiarity among the elderly and their caregivers.

Performance expectancy and effort expectancy were significant predictors of emotion, which was in line with the research of Gursoy et al. (2019), Chi et al. (2023), and Lin et al. (2020). These two relationships derived from the AIDUA model were proven to be effective during AI companion robot adoption. Once users realise the benefits and utility of AI companion robots and the fact that these smart robots are easy to use, their acceptance will rise. For policymakers, they should develop strategies to educate the public about the function and benefits of AI companion robots. For instance, public awareness about robots’ capabilities can be raised through advertising campaigns, public lectures, or online resources. These initiatives can help shift users’ attitudes and increase adoption willingness among those who are unsure or indecisive. For designers, they can try to simplify the user interface of companion robots and offer a favourable interactive experience to improve usability. For senior citizens, the interface of a robot can be designed with large, easily readable fonts, high-contrast colour schemes, and more intuitive navigation. Moreover, some elderly people have significant physical limitations and cannot interact with companion robots smoothly. Hence, designers should consider more appropriate interactive modes for elderly users, such as voice interaction, gesture interaction, and touch interaction.

Our research also contributed to the existing literature by verifying how perceived risk affects users’ affective attitudes. This factor is an extra factor that extends the traditional AIDUA model. Consistent with previous evidence, people who perceive AI companion robots as risky will be less likely to adopt the technology (Seo & Lee, 2021; Al-Adwan et al., 2023). As an emerging technology, AI companion robots remain uncertain and immature at present. To address the issue, policymakers can establish robust regulatory frameworks that govern the ethical use of AI and robotics technologies. Related issues, such as data protection, personal privacy, and safety standards concerning AI companions, should be addressed in these regulations. Moreover, developers and interactive designers should make some attempts to lower risks through robot design. For example, users can be allowed to customise their interaction levels with the robot according to their comfort zone. When users can decide whether the robot performs more passive tasks (like reminding them of medications) or more active roles (such as initiating conversations). This design approach can enhance autonomy and facilitate a sense of control among elderly users (Coghlan et al., 2021). Meanwhile, robots should be designed to ensure transparency during operation. For robot developers, they should inform users how the robot works, what data it collects, and how the data are used during adoption.

We found that emotion could play a noteworthy role in affecting users’ willingness and objection to using AI companion robots. This relationship derived from the AIDUA model was verified in the context of AI companion robot adoption, which coincides with several prior studies (Li et al., 2024; Ma & Huo, 2023; Chi et al., 2023). As a core variable in the outcome stage, “emotion” reflects users’ affective attitude toward AI companion robots, which is determined by three factors: performance expectancy, effort expectancy, and perceived risk. In practical situations, researchers should focus on boosting positive emotions and inhibiting negative emotions by regulating the three determinants.

Finally, we found that gender, age, and educational level do not moderate the relationship between individual characteristic variables and performance expectancy, effort expectancy, and perceived risk. This suggests that elderly individuals share common psychological pathways when evaluating AI companion robots, regardless of gender, age subgroup (e.g., young-old vs. old-old), or educational attainment. For companion robot designers and developers, they should prioritise features that enhance the usability and ease of use of AI companion robots for all elderly users, rather than segmenting by gender, age, or education. Moreover, designers can consider identifying universal priorities for elderly people (e.g., safety and social connection) during the user research. For instance, they can integrate the universal design or inclusive design principles into the companion robot design (Steinfeld & Maisel, 2012).

## 7. Conclusions

In the context of accelerating global ageing, AI companion robots offer an innovative solution to enhance the well-being of elderly individuals by providing emotional support and assisting with daily living activities. Despite these potential benefits, many senior citizens feel redundant or even averse towards adopting such technologies, highlighting a significant barrier to their acceptance. Understanding the factors influencing this acceptance remains limited. To address this gap, this paper aims to construct a theoretical model that elucidates the mechanisms underlying elderly individuals’ willingness to adopt AI companion robots. Drawing from the AIDUA model, our framework specifically targets individual differences in technology adoption. We conducted an empirical study using an SEM approach to test the proposed model. The research findings were discussed to provide guidelines for different stakeholders concerning AI companion robot adoption. The theoretical implications and practical implications of this paper were then discussed.

This study makes several significant contributions to the existing literature. First, this article contributed to the existing knowledge of technology adoption by clarifying the multi-stage decision of elderly users’ acceptance of AI companion robots. Eight key variables that influence elderly individuals’ usage and willingness of AI companion robots were identified. Given that companion robots will be a critical driving force to enhance the quality of life for the elderly and assist with their daily living activities, it is necessary to examine the influential factors of their acceptance. Second, the proposed model was an extension of AIDUA. Based on an SEM analysis, we validated the adaptability and practicality of the AIDUA model in the context of companion robots. Third, unlike prior studies that emphasise robot anthropomorphism or cultural context, our study attempts to reveal the impact of individual differences on users’ decision-making processes. In the primary appraisal stage, three individual characteristic variables (technology optimism, innovativeness, and familiarity) were put forward as prerequisites for other variables. The results can guide stakeholders of companion robots from a novel perspective. Moreover, we added the variable “perceive risk” in the secondary appraisal stage, which describes users’ concern about potential harmfulness in the technology adoption process. The research findings indicated that risk perceptions would raise elderly individuals’ negative affective attitudes toward companion robot adoption. Hence, this study extends the applicable service context of the Technology Readiness Index.

In practical situations, the research findings offer novel insights for stakeholders of AI companion robots. For robot designers and developers, the findings can guide them to focus on features that are desired by users, such as aesthetic appeal design, interaction modes design, and marketing strategies design. Moreover, they need to consider more privacy and ethical issues and attempt to address these problems when designing new generations of products. For elderly individuals, better-designed companion robots can offer healthcare services, daily work assistance, and emotional support. For community workers, our study can provide guidelines for them concerning the spread of companion robots among communities. For policymakers, the findings can guide them in formulating regulatory frameworks and launching public campaigns. A wide adoption of companion robots can ease the pressure on elderly care and maintain stable, productive family units. For technology enterprises, a growing market drives continuous improvement and innovation, pushing the boundaries of what these robots can offer and paving the way for advanced models that cater to evolving user needs.

However, this study has several limitations. First, our research mainly focused on elderly individuals’ acceptance of AI companion robots, overlooking other age groups such as children, adolescents, and young adults. While the elderly population could benefit from the adoption of AI companion robots, these smart devices have the potential to be suitable for other age groups. In effect, AI companion robots that target children or adolescents have emerged in the Chinese market. Second, our study did not consider the impact of cultural context in shaping attitudes toward AI companion robots. Cultural values, such as collectivism in China or individualism in Western societies, can profoundly affect perceptions of technology in healthcare and family dynamics. For instance, the Japanese tend to prioritise the emotional experience associated with human–computer interactions (Li et al., 2024).

In this case, future researchers can address these gaps through targeted investigations. First, researchers can expand the scope to include diverse age groups, and comparative studies can be made to clarify how users’ acceptance of AI companion robots varies with age. In addition, they can conduct longitudinal studies to further track how user perceptions evolve over time as individuals interact with AI companion robots. Second, researchers can carry out cross-cultural analyses to understand how sociocultural norms influence adoption. For example, comparative studies between collectivist societies (e.g., China) and individualist societies (e.g., the U.S.) can be made to reveal the impact of cultural contents on users’ adoption willingness.

## Figures and Tables

**Table 1:** Sample characteristics.

Attribute	Value	Frequency	Percent
Gender	Male	250	55.3%
	Female	202	44.7%
Age	60–64	103	22.8%
	65–69	134	29.6%
	70–74	134	29.6%
	75–79	63	13.9%
	80 or above	18	4.0%
Educational level	Under primary school	89	19.7%
	Junior high school	111	24.6%
	High school/technical secondary school	119	26.3%
	Diploma	90	19.9%
	Undergraduate or above	43	9.5%
Family Monthly income (RMB)	Under 3499	63	13.9%
	3500–6999	135	29.9%
	7000–10,499	145	32.1%
	10,500–13,999	83	18.4%
	Over 14,000	26	5.7%

**Table 2:** Reliability and validity of constructs.

Constructs	Items	Standardised Factor Loading	Average Variance Extracted (AVE)	Composite Reliability (CR)
Familiarity (FAM)	FAM1	0.853	0.721	0.912
	FAM2	0.893		
	FAM3	0.839		
	FAM4	0.810		
Technology optimism (TO)	TO1	0.854	0.690	0.870
	TO2	0.779		
	TO3	0.857		
Innovativeness (INN)	INN1	0.810	0.677	0.863
	INN2	0.813		
	INN3	0.845		
Performance expectancy (PE)	PE1	0.901	0.730	0.915
	PE2	0.859		
	PE3	0.815		
	PE4	0.840		
Effort expectancy (EE)	EE1	0.854	0.730	0.916
	EE2	0.832		
	EE3	0.860		
	EE4	0.872		
Perceived risk (PR)	PR1	0.898	0.762	0.928
	PR2	0.893		
	PR3	0.848		
	PR4	0.852		
Emotion (EMO)	EMO1	0.809	0.709	0.924
	EMO2	0.874		
	EMO3	0.863		
	EMO4	0.813		
	EMO5	0.848		
Willingness to use (WTU)	WTU1	0.830	0.710	0.880
	WTU2	0.858		
	WTU3	0.840		
Objection to use (OTU)	OTU1	0.877	0.720	0.885
	OTU2	0.837		
	OTU3	0.831		

**Table 3:** Correlation matrix and the square root of the AVE.

Construct	AVE	FAM	TO	INN	PE	EE	PR	EMO	WTU	OTU
FAM	0.721	(0.849)								
TO	0.690	0.168 **	(0.831)							
INN	0.677	0.098	0.344 ***	(0.823)						
PE	0.730	0.107 *	0.254 ***	0.293 ***	(0.854)					
EE	0.730	0.144 **	0.288 ***	0.295 ***	0.222 ***	(0.854)				
PR	0.762	−0.281 ***	0.127 *	0.127 *	0.181 ***	0.058	(0.873)			
EMO	0.709	0.175 **	0.302 ***	0.316 ***	0.227 ***	0.299 ***	−0.113 *	(0.842)		
WTU	0.710	0.021	0.206 ***	0.254 ***	0.257 ***	0.226 ***	0.132 *	0.224 ***	(0.843)	
OTU	0.720	0.042	0.123 *	−0.028	0.116 *	0.070	0.089	−0.222 ***	0.121 *	(0.849)

Note: * *p* < 0.05; ** *p* < 0.01; *** *p* < 0.001; the square root of AVE is on the diagonal.

**Table 4:** Hypothesis testing.

Hypotheses	Relationships	Standardised Coefficient	T-Value	*p*-Value	Supported
H1	TO → PE	0.179	2.961	0.003	Supported
H2	TO → EE	0.204	3.674	0.000	Supported
H3	INN → PE	0.239	4.060	0.000	Supported
H4	INN → EE	0.225	3.920	0.000	Supported
H5	FAM → EE	0.088	1.658	0.097	Not supported
H6	FAM → PR	−0.279	−5.360	0.000	Supported
H7	PE → EMO	0.203	3.787	0.000	Supported
H8	EE → EMO	0.271	5.185	0.000	Supported
H9	PR → EMO	−0.161	−3.409	0.001	Supported
H10	EMO → WTU	0.230	4.325	0.000	Supported
H11	EMO → OTU	−0.218	−3.911	0.000	Supported

**Table 5:** Multigroup analysis (gender).

Path Direction	Male	Female	Sig. Diff.
TO → PE	0.176 *	0.175 *	0.001
TO → EE	0.234 **	0.154	−0.166
INN → PE	0.184 **	0.350 ***	0.079
INN → EE	0.219 **	0.285 **	−0.066
FAM → EE	0.023	0.159 *	−0.136
FAM → PR	−0.221 **	−0.360 ***	0.139

Note: * *p* < 0.05; ** *p* < 0.01; *** *p* < 0.001.

**Table 6:** Multigroup analysis (age).

Path Direction	Low-Level Group	High-Level Group	Sig. Diff.
TO → PE	0.236 **	0.139	0.097
TO → EE	0.287 ***	0.117	−0.094
INN → PE	0.214 **	0.308 **	0.170
INN → EE	0.231 **	0.224 *	0.007
FAM → EE	0.039	0.138	−0.098
FAM → PR	−0.266 ***	−0.319 ***	0.053

Note: * *p* < 0.05; ** *p* < 0.01; *** *p* < 0.001.

**Table 7:** Multigroup analysis (education).

Path Direction	Low-Level Group	High-Level Group	Sig. Diff.
TO → PE	0.221 **	0.143	0.077
TO → EE	0.290 ***	0.135	−0.139
INN → PE	0.197 *	0.336 ***	0.154
INN → EE	0.186 *	0.305 **	−0.120
FAM → EE	0.044	0.120	−0.076
FAM → PR	−0.240 **	−0.325 ***	0.085

Note: * *p* < 0.05; ** *p* < 0.01; *** *p* < 0.001.

## Data Availability

The data presented in this study are available on request from the corresponding author.

## Appendix A

**Table A1:** Constructs and items.

Constructs	Items and Descriptions	References
Familiarity (FAM)	FAM1: I am familiar with the related information and knowledge of AI companion robots. FAM2: I am familiar with brands and products of AI companion robots. FAM3: I am familiar with services provided by AI companion robots and their functions. FAM4: I am familiar with how to operate AI companion robots.	(J. Liu et al., 2023)
Technology optimism (TO)	TO1: Products and services that leverage the newest technologies are much easier to use. TO2: I prefer to use the most cutting-edge technology available. TO3: Technology enhances the efficiency of my work.	(Álvarez-Marín et al., 2023)
Innovativeness (INN)	INN1: If I find out that there are new technologies, I seek avenues to test them. INN2: Among my peers, I usually take the lead in trying these new technologies. INN3: I like to experiment with new technologies.	(Álvarez-Marín et al., 2023)
Performance expectancy (PE)	PE1: I would find using AI companion robots useful in daily life. PE2: Using AI companion robots would help me accomplish things more quickly. PE3: Using AI companion robots has increased my productivity. PE4: AI companion robots would increase my chances of achieving things that are important to me.	(Venkatesh et al., 2012)
Effort expectancy (EE)	EE1: Learning how to use AI companion robots would be easy for me. EE2: My interaction with AI companion robots would be clear and understandable. EE3: I would find using AI companion robots easy. EE4: It would be easy for me to become skilful using AI companion robots.	(Venkatesh et al., 2012)
Perceived risk (PR)	PR1: The risk of using AI companion robots is high. PR2: The likelihood of unexpected problems with using AI companion robots is high. PR3: The degree of uncertainty related to using AI companion robots is high. PR4: Overall, the possibility of adverse consequences associated with using AI companion robots is high.	(Esmaeilzadeh, 2020)
Emotion (EMO)	EMO 1: Bored-relaxed EMO 2: Malancholic-contented EMO 3: Despairing-hopeful EMO 4: Unsatisfied-satisfied EMO 5: Annoyed-pleased	(Lu et al., 2019)
Willingness to use (WTU)	WTU1: I am willing to use AI companion robots. WTU2: I feel happy to interact with AI companion robots. WTU3: I am likely to interact with AI companion robots.	(Lu et al., 2019)
Objection to use (OTU)	OTU1: I prefer human contact in service transactions. OTU2: I need emotional exchange during service transactions. OTU3: Interaction with AI companion robot lacks social contact.	(Lu et al., 2019)

**Table A2:** Summary of experts’ views and suggestions.

No.	The Original Questionnaire	The Experts’ Suggestions	Authors’ Response
1	…The AI companion robot can interact with the elderly through voice, images, and other means to provide emotional support and companionship.	Use examples to introduce the type and function of AI companion robots directly.	The revision is as below: …The AI companion robot can interact with the elderly through voice, images, and other means to provide emotional support and companionship. Datou Aliang elderly companion robot, “Xiaoli” elderly companion robot, UBTECH Health Care Robot Series. Here is the figure of the companion robot in this study.
2	/	Highlight the anonymity and informed consent form at the beginning of the questionnaire.	We have added instructions for anonymous filling and an informed consent form at the beginning of the questionnaire.
3	Income (RMB)	Please give a more specific explanation of the variable: income.	We have used “Family monthly income (RMB)” to replace the original “income”.
4	Family monthly income (RMB): Under 3500; 3500–7000; 7000–10,500; 10,500–14,000; Over 14,000	The values at both ends of the interval should not be repeated.	The revision is as below: Family monthly income (RMB): Under 3499; 3500–6999; 7000–10,499; 10,500–13,999; Over 14,000
5	/	Translate attributive clauses should conform to Chinese reading habits, such as “Products and services that leverage the newest technologies are much easier to use”. Please check all sentences involving this issue.	We have modified all sentences involving the translation of attributive clauses to conform to Chinese reading habits.
6	/	When measuring one dimension of the variable “emotion”, such as “Bored-relaxed”, you should clearly describe the options corresponding to scores 1 to 5.	We have provided particular explanations for this question and described each option corresponding to scores 1 to 5 separately.
7	/	Please use the back-translation method or AI tools to check if the English-to-Chinese translation is accurate.	We have used the back-translation method to check the accuracy of translation.
